# Bioinformatic Methodologies in Assessing Gut Microbiota

**DOI:** 10.3390/microbiolres15040170

**Published:** 2024-12-03

**Authors:** James Douglas Fox, Austin Sims, Morgan Ross, Jeffery Bettag, Alexandra Wilder, Dylan Natrop, Alison Borsotti, Sree Kolli, Shaurya Mehta, Hema Verma, Kento Kurashima, Chandrashekhara Manithody, Arun Verma, Ajay Jain

**Affiliations:** 1 Department of Pediatrics, Saint Louis University, St. Louis, MO 63103, USA; 2 Medical College of Wisconsin-Green Bay, De Pere, WI 54115, USA; 3 SLU College for Public Health and Social Justice, Saint Louis University, St. Louis, MO 63104, USA

**Keywords:** microbiome, intestinal microbiology, bioinformatics, whole-genome sequencing, 16S rRNA sequencing, shotgun sequencing, gastrointestinal, gut microbiota

## Abstract

Bioinformatic methodologies play a crucial role in the assessment of gut microbiota, offering advanced tools for analyzing complex microbial communities. These methodologies involve high-throughput sequencing technologies, such as 16S rRNA gene sequencing and metagenomics, which generate vast amounts of data on microbial diversity and functional potential, as well as whole-genome sequencing, which, while being more costly, has a more expansive potential. Bioinformatics tools and algorithms process these data to identify microbial taxa and quantify and elucidate their roles within the microbiome. Advanced statistical and computational models further enable the identification of microbiota patterns associated with various diseases and health conditions. Overall, bioinformatic approaches are essential for deciphering the complexities of gut microbiota so that, in the future, we may be able to discover treatments and technologies aimed at restoring or optimizing the microbiome. The goal of this review is to describe the differences in methodology and utilization of 16S versus whole-genome sequencing to address the increased understanding of the role that the gut microbiome plays in human physiology and pathology.

## 1. Introduction

The implications of microbiota in the gastrointestinal tract have become increasingly important in understanding the symbiotic relationship between bacteria, human health, and disease. There is growing evidence that the gut microbiome influences multiple organ systems and processes and has a significant role in nutrient, vitamin, and mineral absorption, processing, and utilization by the human gut [1,2]. The gut microbiome (which not only includes the collection of all organisms but also their theater of activity, which, in this case, is the human gut) evolves together with the host’s innate and adaptive immunity and is an effective first line of defense against enteropathogens. It also assists in processing antigens by activating non-antigen-specific pattern recognition receptors [3-5]. As expected, alteration in the gut microbiome composition also modulates the immune system—some people become more susceptible to infection based on alterations to their gastrointestinal flora [6-8]. Various inflammatory, metabolic, and neurodegenerative disorders have also been linked to the gut microbiota [1,2]. Given the human microbiome’s role in maintaining health, it is critical to better comprehend the balance of the gut microbiota and its relevance to normal function and various pathologies. Metagenomics, which is defined as the application of genomics techniques to study complex communities of organisms within their natural environment, is the main field in which these complex and intertwined aspects are studied.

**Figure 1.**
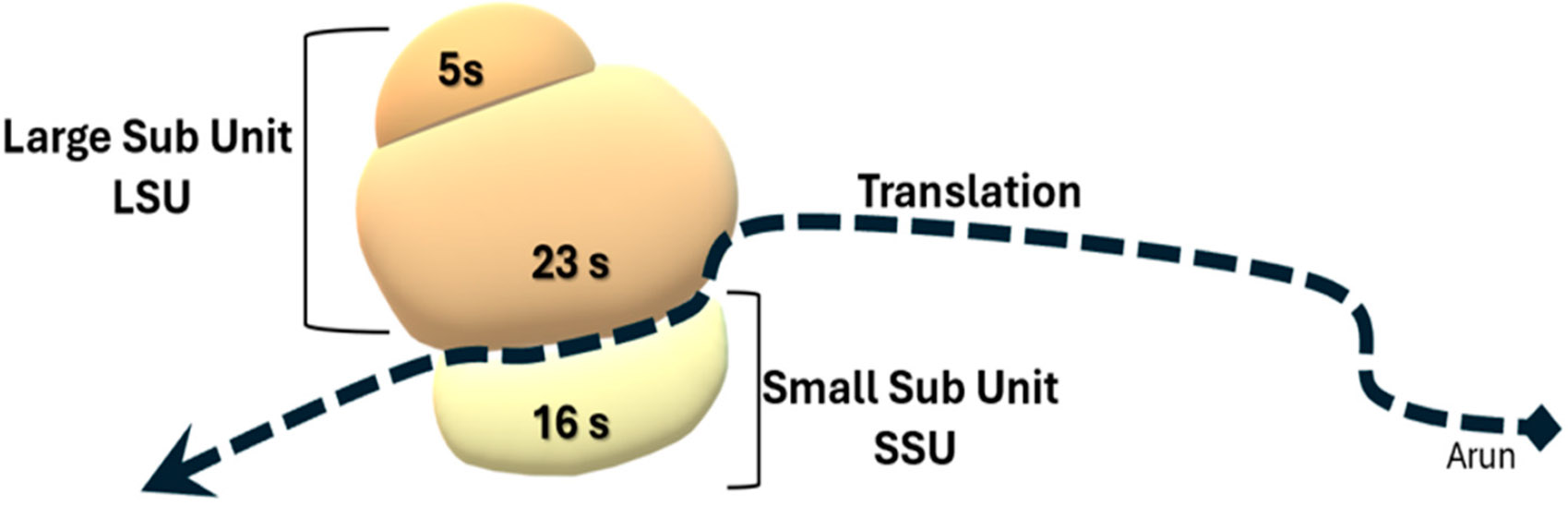
Components of the prokaryotic ribosome, depicting the subunits and the translation process.

**Figure 2.**
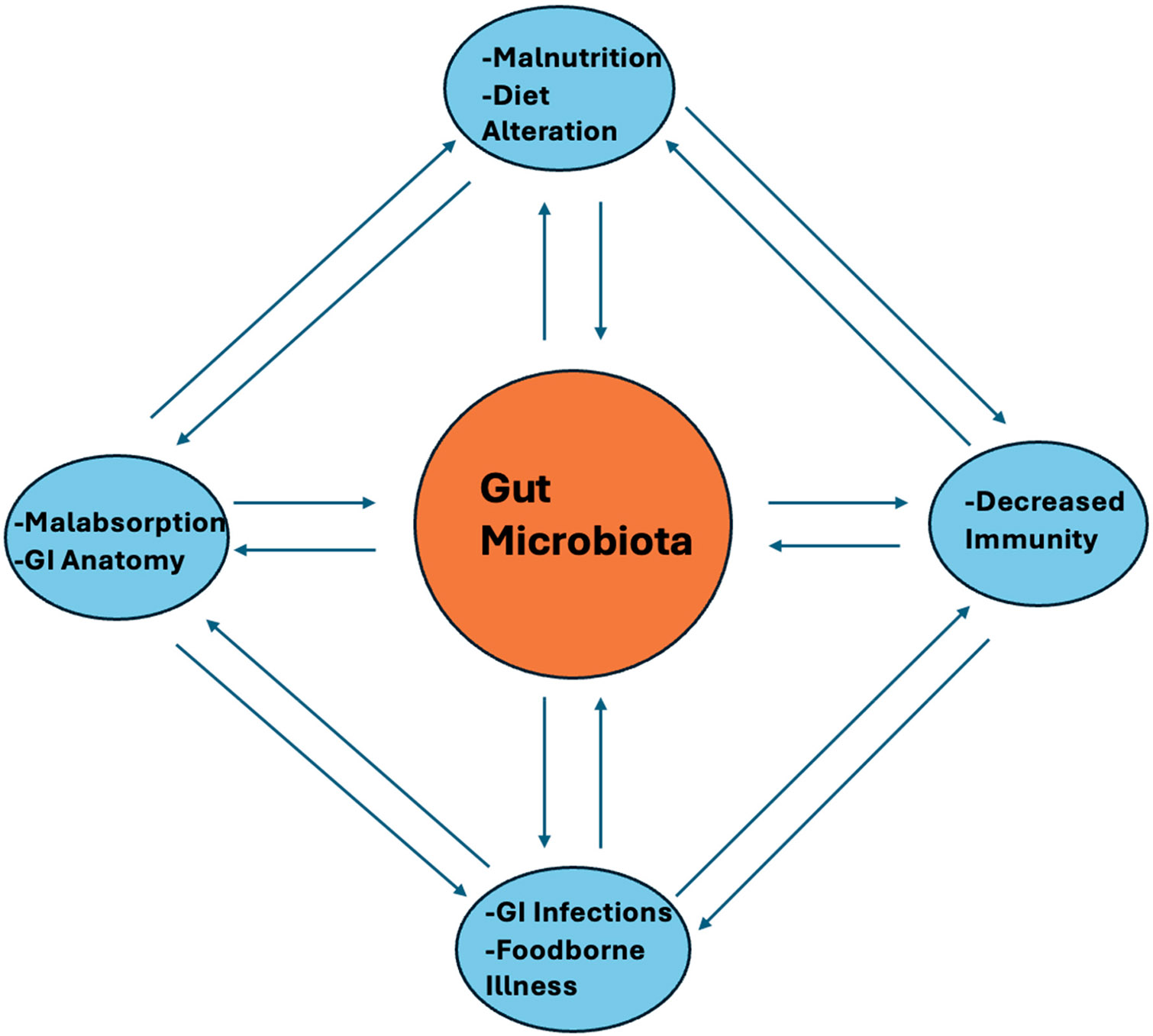
Complex multidirectional interactions of gut microbiome with major influencing factors of the unique host genotype and core modulation by the innate and adaptive host immunity.

The two main methodologies for assessing the gut microbiome are whole-genome shotgun (WGS) sequencing and 16S rRNA (16S RNA) sequencing. 16S rRNA stands for 16S ribosomal ribonucleic acid (rRNA), where S (Svedberg) is a unit of measurement (sedimentation rate). This rRNA is an important constituent of the small subunit (SSU) of prokaryotic ribosomes [9], as seen in Figure 1. 16S RNA was first discovered and described for identification of prokaryotic life forms by Carl R. Woese and George E. Fox in a pioneering article in 1977 [10]. Expanding on their work,16S RNA sequencing was used for the taxonomic classification of prokaryotes for the first time in 1985 [11]. 16S RNA sequencing has been the most commonly used method until recently and consists of amplifying a 16S rRNA region via PCR. Variable regions of the 16S RNA sequences are then detected in order to identify different species and the composition of the gut microbes [12]. On the other hand, WGS sequences the organisms’ entire genome, providing a more comprehensive analysis of the microbiome [13]. Over time, the availability of different bioinformatic methodologies has changed as technologies have improved, and choosing the most appropriate methodology is very specific to what a certain laboratory is studying. There are many advantages and disadvantages of both methods, including but not limited to cost and time, applications, resolution and specificity, and sample requirements. Decisions about which methodology is appropriate depend on these factors and what is needed from the analysis of the microbiome [14]. There is a paucity of recent research specifically comparing and contrasting 16S RNA and WGS sequencing in assessing the gut microbiome. This review aims to provide a clearer and more concise overview of these sequencing technologies and how best they can be used in furthering our understanding of the implications of the gut microbiome (Figure 2).

## 2. Research Methodology

**Scheme 1.**
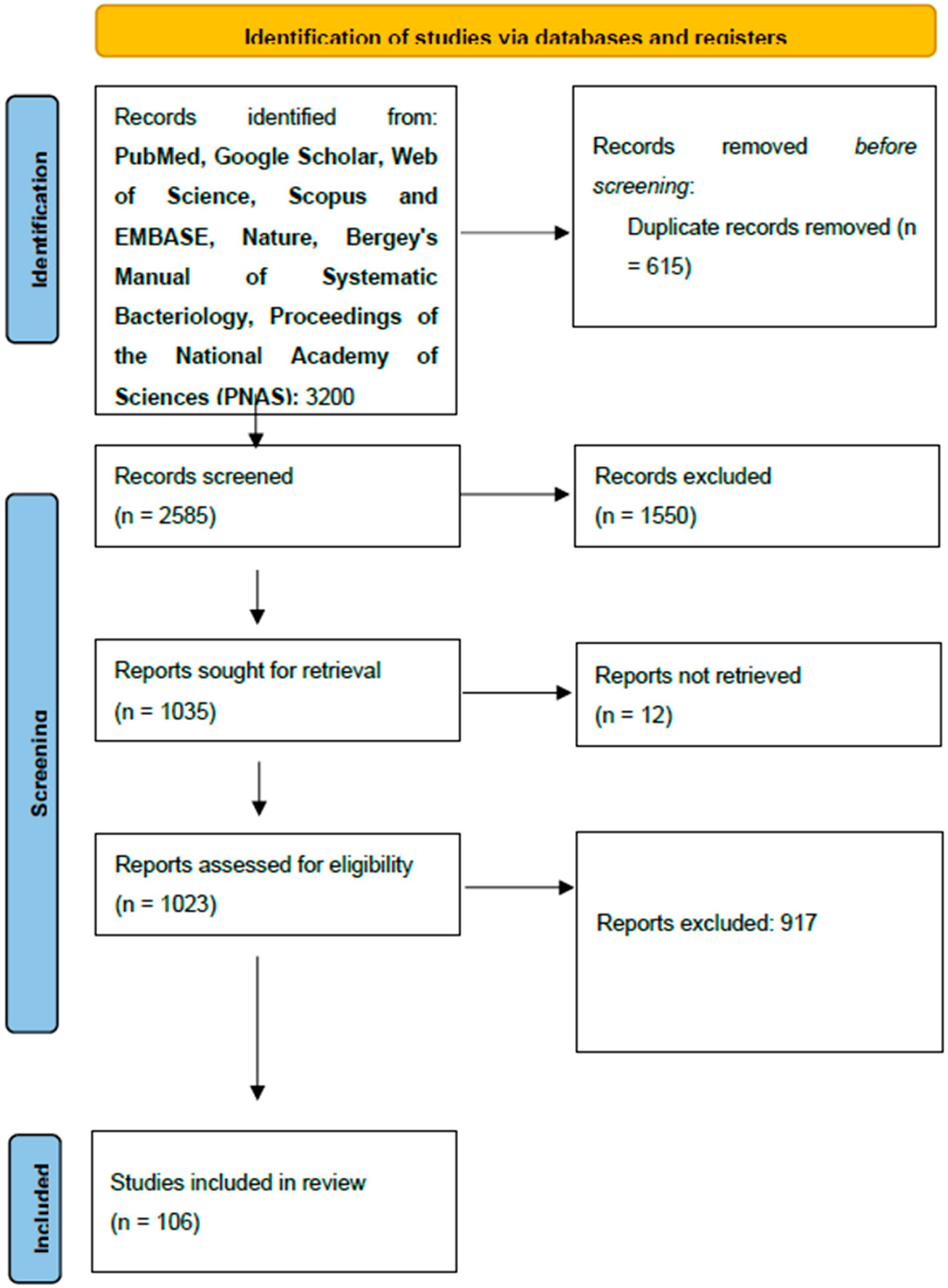
Source: Page MJ et al. BMJ 2021;372:n71. https://doi.org/10.1136/bmj.n71 [15]. This work is licensed under CC BY 4.0. To view a copy of this license, visit https://creativecommons.org/licenses/by/4.0/ (accessed on 13 November 2024).

For the purpose of this systematic review, we utilized standard operating principles as outlined in the PRISMA [Preferred Reporting Items for Systematic Reviews and Meta-Analyses] reporting guidelines. (Scheme 1) [15].

Sources utilized were PubMed, Google Scholar, Web of Science, Scopus, EMBASE, Nature, Bergey’s Manual of Systematic Bacteriology, and Proceedings of the National Academy of Sciences (PNAS).

Search Method:

- Keyword Search: We used specific keywords and phrases, utilizing the MeSH feature of PubMed, such as “16S rRNA gene sequencing”, “Whole-genome sequencing”, “Shotgun sequencing”, “gut microbiota”, “dysbiosis”, “microbiome”, “intestinal microbiology”, “bioinformatics”, and “gut microbiota”.
- Boolean Operators: Operators like AND, OR, and NOT were used to refine search results (e.g., “gut microbiota AND16S RNA sequencing”).
- Filters Used: Articles in the English language were used, and the rest were discarded. Abstracts were reviewed to select studies that met the inclusion criteria. Only articles that had the full text available were utilized for review and citation. Any duplicates were then removed.
- Snowballing: We reviewed the references in key articles to identify additional studies that may be relevant to our research topic.
- Final Study Selection: At least two authors reviewed the publications independently to assess the quality of included sources, and any disagreements were resolved by discussion/third author review.

Years Covered in Search:

- Our review included publications from the last 24 years to ensure that the most current and relevant research was considered. For foundational studies, including the discovery and further development of specific methods (e.g., the introduction of 16S rRNA sequencing), we included earlier landmark papers dating back to 1977. Utilizing the snowballing method, highly cited papers were sought after reviewing the bibliography of the relevant published literature in the hope of citing the source papers in most instances.

Inclusion Criteria:

- Published resources in English.
- Resources with full text.
- Studies with abstracts relevant to the application of Bioinformatics in gut microbiota research.

Exclusion Criteria:

- Resources published in a language other than English.
- Resources without available full text.
- Studies focusing on topics irrelevant to our research objective.

## 3. Development of the Microbiome

Human microbiome development starts in utero in pregnancy, with the current literature suggesting the fetus has its own microbiome and possibly engages in transfer between mother and fetus [16]. The microbiome is then modified during childbirth and infancy, strengthened by breastfeeding, and established by weaning and diet transition in the first three years of childhood [17]. It gets altered, regulated, and fine-tuned by the “Sibling Effect”; social, environmental, and geographical effects; and matures till adulthood. The host gut microbiome is affected by myriads of variables, including host genotype, environment, xenobiotics (drugs and environmental toxins), stress levels, etc. Socioeconomic status, including income level, social crowding, diet, and education, influence the development of gut bacterial diversity [3,18]. This Exposome [3,18] (“the cumulative measure of environmental influences and associated biologic responses throughout the life span, including exogenous exposures and endogenous processes”) is variable in communities and individuals separated in time and geography. However, in closely related communities and individuals, it bears resemblance. This Sociobiome [3,18] can be defined as the microbiome composition of a geographic region or neighborhood as a result of exposure to similar socioeconomic and environmental factors, which facilitate an exposure with analogous characteristics that shape the individual microbiome into remarkable resemblance. This has important implications for the development of innate and adaptive immunity and allergic, immune, and neoplastic diseases. Any changes in the above factors, especially the exposome or sociobiome, can cause derangement in the usual composition of microbial richness and diversity, called dysbiosis [3,18].

Attempts have been made to restore the dysbiosis (deranged microbiome). So far, therapeutic trials of prebiotics, probiotics, and dietary interventions have been tried to restore bacterial diversity in the gut, but with inconsistent results. Fecal transplant has also been tried in some studies, with promising results in select cases [19-21].

An exhaustive assessment of the prevalent sociobiome and any dysbiosis is essential, has important diagnostic and therapeutic implications to develop tailormade therapies, and can permit targeted health policies for regions with varying realities and issues, instead of broad/blind interventions.

A universal marker for the relationship between gut microbiota and pathophysiological processes does not exist. But we can derive the trends in an individual and in the population by evaluating diet and lifestyle, unraveling the exposome, and by genetic analysis [3,18].

## 4. 16S Sequencing

### 4.1. Description and Methodology

Given the growing knowledge in the last two decades of the widespread impact of the microbiome on various pathologies across all organ systems, an efficient, effective, and accurate way to sequence the microbiome has been investigated rigorously. One such method, as previously mentioned, is 16S RNA genome sequencing. Prior to 16S sequencing, culture-based technology was used and was expensive, time consuming, and inaccurate [22], only lending gross information on species and relationships to other microbiota, missing out on key details that differentiate one from another and possibly leading to significant human pathologies, such as multiple sclerosis, Parkinson’s, blood pressure, stroke, and GI disorders, amongst others [23].

Before comparing 16S RNA sequencing and WGS sequencing, we will discuss the methodology of 16S technology. In order to attach scientific names to small organisms within the human body, the field of clinical microbiology needed a way to differentiate new strains that would frequently be identified. Historically, this was achieved using different manuals or collections of information on morphologic and phenotypic characteristics of different strains, and microbiologists would attempt to fit a newfound strain into predetermined categories [24,25]. Logically, however, this would lead to frequent errors in classifications or newfound strains that were between such finite descriptions. As a result, significant differences were often seen between different researchers across many fields and labs.

**Figure 3.**
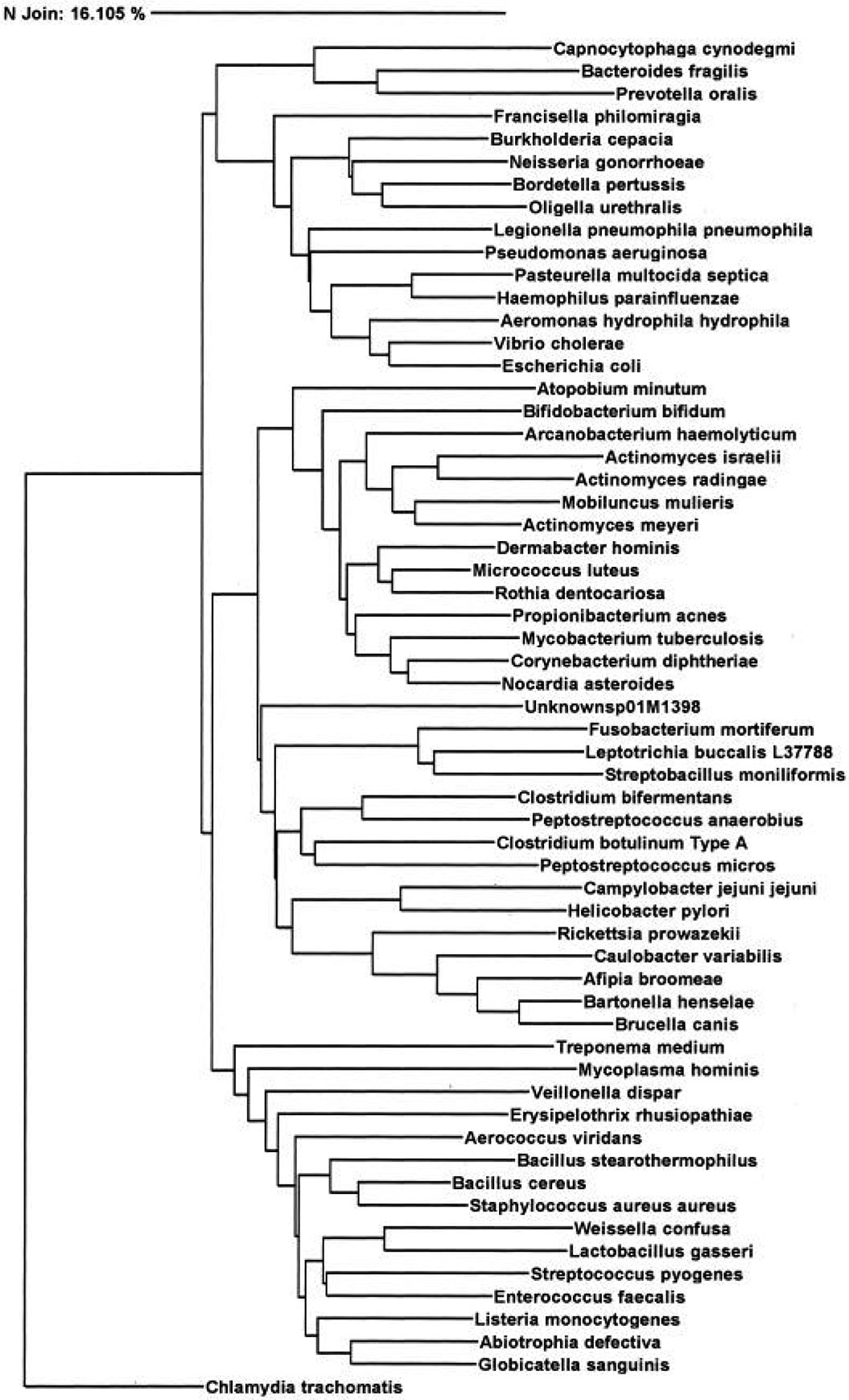
16S rRNA sequencing dendrogram deriving phylogenetic relationship (borrowed with permission from the American Society for Microbiology 1988).

By the 1980s, it was found that an evolutionary part of the genome of all forms of life was particularly stable from mutation over time and could be compared to understand the phylogenetic relationship of species, and that piece was the 16S RNA sequence for bacteria. The reason for the evolutionary stability of the 16S RNA piece is that it codes for a necessary component for bacterial life, a subunit of the ribosome, which produces proteins for the organism [26]. Across all bacteria, this segment stays generally consistent in length, and although the mutation rate is unknown, it is particularly slow overall despite some ‘hot spots’ in variable regions of the sequence. The 16S RNA sequence is known to be about 1550 base pairs long, providing adequate length to disclose interspecies variation as well, down to the level of the genus (Figure 3). The 16S RNA sequence, like many others in genome sequencing, is composed of both variable and conserved regions to allow for such comparisons and differentiation [27]. Additionally, studies have shown differentiation with 16S sequencing down to the level of the species and subspecies, or subgroups within a single species showing distinct genetic variation yet retaining breeding capabilities [28].

**Figure 4.**
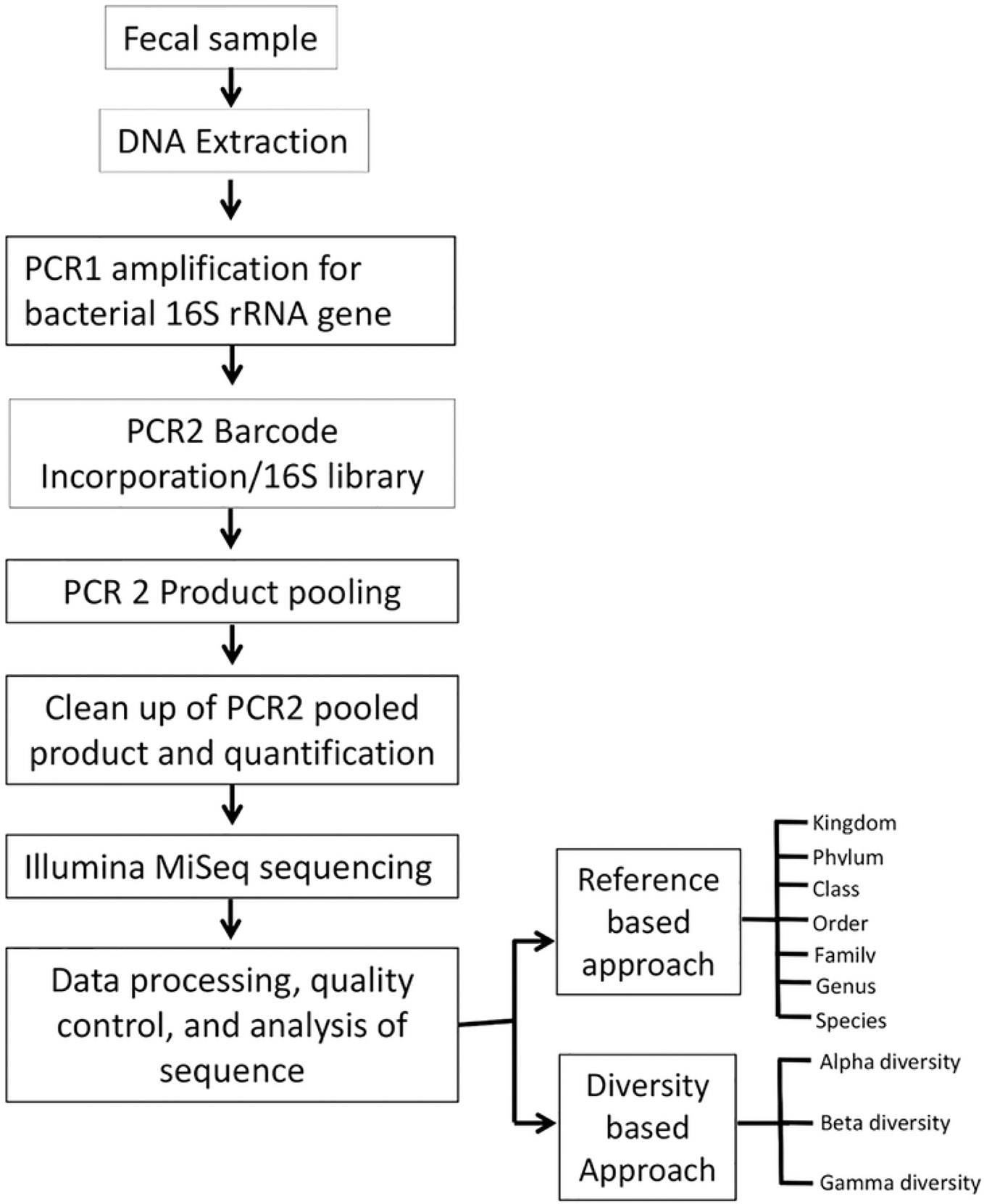
Flow diagram of gut microbiome sequencing. Reproduced with copyright permission.

Interestingly, although 16S RNA sequencing follows the rough step-by-step process of all sequencing (cut DNA into fragments with primer to isolate, duplicate, and sequence with computerization), there has been no standardized process specific to 16S RNA, despite the fact several have been proposed [23,29]. Although there are indications to sequence the whole 1550 base pairs or a shorter segment, most of the difference in protocol stems from rapid technological advancement. Different products over the past decades at various costs have influenced protocol. For instance, only forward sequencing is cheaper, but lower quality and older kits could only sequence 150–300 base pairs at a time, requiring the merging of two reads individually. Now, less expensive kits have advanced to sequence up to 600 base pairs at one time (e.g., Illumina HiSeq, San Diego, CA, USA). However, both of the aforementioned options are small kits for lower profile or longer drawn-out work, and currently, technology can accomplish much more at laboratories with more resources and equipment. Generally, shorter segments, such as the 500 base pair segment commonly used, are cheaper and more useful for interspecies variation and diagnosis, while whole 16S sequencing provides information on subspecies variation [30]. One such protocol, proposed by Shahi et al. in 2019 for gut microbiota sequencing, suggests that the protocol be standardized, as shown in Figure 4 below.

### 4.2. What Does 16S Sequencing Help Investigate?

16S RNA sequencing within metagenomics has diverse applications in studying the gut microbiome, ranging from basic research to clinical application. Historically, bacteria were identified through culture, which only identified a subset of bacteria as many do not grow well on common media. The 16S RNA sequencing method allows for the identification of a greater number of bacterial communities in the gut microbiome as an effective alternative to culture [31]. Notably, while the 16S RNA gene is a marker in bacteria and archaea, it does not help in the identification of fungi, viruses, or eukaryotes. It is used to characterize the composition of bacterial communities in different environments, such as the human gut, allowing for the identification and classification of bacteria, as well as providing information on the diversity and abundance of different taxa. With enhanced characterization of the gut microbiome comes more potential for clinical applications. Accurate tracking and identification of pathogens in hospital-acquired infections, especially those resistant to antibiotics, is incredibly important in epidemiology and clinical microbiology [32]. Using 16S RNA sequencing can effectively identify bacteria that could not otherwise be cultured, thus enhancing antibiotic stewardship and decreasing the utilization of empiric antibiotics while awaiting culture results [33]. 16S RNA sequencing also has the potential to evaluate the presence and absence of resistance genes and monitor the emergence and spread of pathogens within communities and hospitals [34-36]. However, it is important to note that whole-genome sequencing may be more informative in this role as 16S sequencing often classifies at the genus level rather than specific strains or species, and the distinctions and advantages between each method will be discussed later [37,38]. Moreover, further identification and characterization of the gut microbiome by 16S sequencing may benefit the development of drugs metabolized by the GI microbiome [39]. Bacteria can change the activity, bioavailability, and toxicity of metabolized drugs. Thus, pharmacokinetics and the gut microbiome are intrinsically linked. Altering the microbiome through dietary changes and probiotics can thus be used to enhance medication efficacy and delivery [40]. Vice versa, through the identification of bacteria involved in a drug’s metabolism, medications can be designed to target enzymes of specific bacteria to inhibit undesired side effects [40,41]. However, it is notable that studies of the genetic sequencing of the gut microbiome suggest that bacterial composition varies depending on the primer set used and differs between individuals of different geographic origins, ages, genders, and body habitus [37,42-44]. For instance, a study comparing the cecal microbiome of obese and lean mice looked at 5088 bacterial 16S RNA gene sequences and found that obesity significantly alters the gut microbiome [45]. The beneficial impact of implementing treatment based on such bacterial variations in obesity has been elucidated in a variety of animal and human studies [46-48]. Furthermore, evaluating the composition of the gut microbiome through 16S sequencing may help provide clarification on the progression of some diseases. A study compared gut microbiome composition between patients of different stages of multiple sclerosis and healthy controls by 16S RNA analysis and found that changes in some bacterial species were closely associated with the clinical severity of patients [49]. This suggests the need to consider the gut microbiome when considering a patient’s health, development, and nutritional needs and presents a potential opportunity for new treatment strategies.

### 4.3. Efficacy/Accuracy of 16S Sequencing

The potency of this molecular technique’s results hinges upon the targeted approach of characterizing the 16S RNA sequences. From sample collection to sequencing, meticulous handling is crucial to successful results.

The accuracy of 16S RNA sequencing has greatly increased over the past few decades. Compared to its early days, there are much more accurate fragment separation and sequencing deposits [50]. Further refinement of this technique has led to an increase in information dispersed in commercial databases (e.g., MicroSeq, GenBank-EMBL, GreenGenes) [51]. Though these databases have been filtered over the years, it is still possible for sequence comparisons to produce false notions. For example, a study conducted on *Actinomyces* strains found that comparing the clinical strains to those found in MicroSeq showed little variability, indicating good quality strains, while the GenBank’s strain, specifically of *A. gravaenitzii,* contained 25 N’s (base unknown) in a 500 bp sequence. These specific comparisons could initially indicate that *A. gravaenitzii* had greater genetic variation than they had in reality. Due to such differences, 16S RNA sequencing was best suited for initial identification with a priori knowledge for the majority of the 2000s.

Common practice has been to sequence 500 bp of choice from the whole 1500 bp length. Especially regarding clinical bacterial pathogen isolates, the initial 500 bp has been a widely sequenced region. Several bacteria can account for the vast majority of variability being found in this region. One such example is the *Bordetella* group, which accounts for over 60% of total species variability in just this initial region [30]. For the sake of creating an industry standard, a benchmark of 95% similarity in the sequence has been necessitated for classification as the same genus and 97% similarity was required for classification as the same species [52]. Aside from the initial 500 bp region, another more recent hotspot for sequencing has been the V4 region [53]. However, more recent findings have suggested these standards and regions of examination are not sufficient.

Analyses of algorithms utilizing identity cross-validation strategies measuring variability in query searches to reference database entries found accuracy levels below 50% in the V4 region, prompting further discussion on regional sequencing ambiguity [54]. Instead, due to technological innovation such as Circular consensus sequencing (CCS) and quality control measures like denoising processes, we can see more accurate results with 16S RNA sequencing of the whole rRNA unit.

Another study examined nonredundant full-length sequences from GreenGenes and generated silico amplicons incorporating common PCR primer binding sites [55]. These amplicons were then examined to see to what level of accuracy they could accurately classify the species taxon to which they belonged. The researchers observed significant variation in the ability of the subregions to distinguish different and accurate species compared to the full-length 16S sequences. The V4 region found the least accurate results, with 56% of the amplicons unable to correctly match their species origin. In comparison, the whole 16S RNA sequencing found much greater levels of success, with it being reported that almost all samples were able to be correctly matched. The study found analyzing the full V1–V9 regions produced the most accurate results. However, they also concluded that different key regions were better suited for specific types of clinical bacterium, such as the V6–V9 region for sequence classification belonging to the genera *Clostridium* and *Staphylococcus*, the V3–V5 region for *Klebsiella*, and the V1–V3 region for *Escherichia*/*Shigella*.

With the rapidly advancing technology utilized in genomics, the accuracy and efficacy of 16S RNA sequencing have vastly improved. Sequencing technology will continue to advance. For example, the most recent third-generation sequencing platforms now enable high throughput sequencing of reads over 1500 bp, which improves accuracy and the ability to differentiate closely related species. Denoising algorithms are also continuously advancing, eliminating sequencing errors and improving reliability [55]. Furthermore, single-nucleotide polymorphisms (SNPs) have been heavily relied upon to differentiate between different strains of bacteria within the gut microbiome, opening up further avenues for clinical exploration and intervention [56].

### 4.4. Advantages and Disadvantages of 16S Sequencing

There are both advantages and disadvantages when looking at 16S RNA gene sequencing as a strategy for identifying the gut microbiome when compared to other bioinformatic methodologies. Known benefits of 16S RNA sequencing include its ability to identify non-culturable bacterial species, low cost, relatively short run and identification times, and small sample size requirements. On the other hand, identifiable weaknesses of this method include its inability to identify non-bacterial species, as well as its relatively low specificity when it comes to identifying past the genus taxonomy [36]. 16S RNA gene sequencing allows for the identification of non-culturable bacterial species. The use of 16S RNA sequencing as a culture-independent modality allows for better identification of the extensive microbiome, given that it is known that less than 1% of bacteria can be cultured with standard technique methods [57]. When looking at a study performed by Rampini et al., it was found that the sensitivity and specificity of 16S RNA sequencing were 42.9% and 100% for culture-negative bacterial infections, respectively [58]. The use of 16S RNA sequencing is known to be one of the most cost-effective methods. Over the past decade, the cost of genetic sequencing has greatly decreased, which has had a staggering impact on microbiology [59]. 16S RNA sequencing costs have substantially decreased in the past years due to new sequencing instruments, technologies, and improved chemistries. Additionally, since 16S RNA sequencing has been used for many years within the field of microbiology, there are a plethora of extensive databases available for the use of identification following sequencing. This leads to a large benefit when it comes to lower cost, given that library and database identification remains one of the leading effects of cost, as well as accurate identification of organisms [59]. Contrarily, a notable shortcoming of the 16S RNA sequencing method is its inability to identify the entirety of organisms within the given sample. The method is only able to determine bacterial genus/species while not being able to make an identification of fungi, protozoa, or viruses within the sample [57]. While the 16S RNA gene is ubiquitous in bacteria, the gene is not present within non-prokaryotic organisms, as they do not possess a 70S ribosome. Given that only bacteria and archaea may be identified with this method, other methods, including 18S rRNA and internal transcribed spacer rRNA sequencing, must be utilized when attempting to identify fungi or eukaryotes, as seen in [60]. The most evident limitation of 16S RNA sequencing is its inconsistency in the ability to identify beyond the genus level [36,57,61]. There have been numerous studies conducted demonstrating that 16S RNA sequencing is able to identify an organism’s genus >90% of the time while only being able to identify its species 65–83% of the time [61-63]. The reason for this constraint is the limited number of sequences within nucleotide databases; consequently, those species that share similar or close to identical 16S RNA sequences are unable to be differentiated at this time [61]. Given 16S RNA sequencing’s poor discriminatory power in regard to differentiating at the species level, additional DNA-related studies, for instance, reciprocal hybridization reactions, may need to be conducted in order to characterize at the species level [61,62,64]. This can be exhibited by a study conducted by Fox et al., which found that there was a >99.5% 16S RNA sequence similarity between two different species of *Bacillus* (*B. psychrophilus* and *B. globisporus*), but when a DNA reciprocal hybridization reaction was conducted there was found to only be 23–50% relatedness between the two species [64]. Various investigations have noted difficulty identifying specific organisms at a genus and/or species level; these include but are not limited to rapid-growing mycobacteria, Stenotrophomonas, Enterobacteriaceae, Achromobacter, and Actinomyces [61,64]. While 16S rRNA sequencing serves its benefits as being a culture-free, low-cost method, there remain certain disadvantages to the methodology, namely its low specificity in regard to classifying at the species level and inability to identify non-bacterial and archeal organisms. Nevertheless, with continually growing databases, 16S RNA remains a valuable tool in assessing the gut microbiome.

## 5. Whole-Genome Sequencing

### 5.1. Description and Methodology of WGS

Whole-genomic shotgun sequencing within metagenomics can be traced back to two extremely impactful studies, one of which was published in 2004 by Venter et al. [65], and the second was published in 2005 by Tyson et al. [66]. Both studies utilized what was then a new sequencing technique called shotgun sequencing to generate small random fragments of genetic material from all organisms within their given samples [65,66]. These studies showcased that it was feasible to characterize the entire genetic material of microbial communities, which before this point had been extremely difficult. The prior difficulty was mainly due to most microbes being incompatible with cultivation in a laboratory setting, as required for earlier whole-genomic sequencing techniques [67,68]. The Human Microbiome Project (HMP), initiated in 2007 and which lasted until 2016, used the technique and lessons learned from the previously mentioned studies to great effect [69]. The project’s overall objective was to facilitate the characterization of the human microbiome so that researchers and clinicians could better understand how it impacts health and disease pathologies within individuals [70]. Both phases of the HMP, along with the associated MetaHIT project, generated more than 2000 reference genomes and identified over 9.75 million microbial genes in the human gut, respectively [71]. The sequencing technique is now known as whole-genome shotgun (WGS) sequencing and is one of the primary genome sequencing methods used in metagenomics [72]. As previously mentioned, WGS metagenomics allows researchers to indiscriminately sequence all DNA material within a given sample, facilitating a culture and cloning-independent form of analysis [73]. With the entire genetic material of a sample being recovered, WGS sequencing can fully differentiate the complete microbial diversity, including bacteria, archaea, eukaryotes, plasmids, and viruses [72,74].

The methodology of WGS sequencing can be broken down into several main steps, which are as follows: sample collection and preparation, library preparation, DNA sequencing, and, finally, bioinformatics data analysis. Regarding sample collection and preparation, it is important to note that protocols for this step can affect the accuracy of the metagenomic data, and as such, collection and storage methods that are validated for a specific sample type cannot be used for other sample types with full confidence [75]. The key objective of this step is to collect sufficient microbial biomass that represents the diversity of the environment being studied [76]. Once a sufficient sample has been taken it must undergo processing to isolate and extract the DNA contained within it. The exact method for isolating DNA must be chosen based on the sample type, as extraction and isolation can affect the downstream sequencing composition, along with the specific type that will be utilized later [75,77]. The two main extraction methods used are mechanical and chemical lysis, with mechanical lysis often being considered superior [78].

Library preparation and DNA sequencing occur next and are chosen based on several variables such as cost, availability of required materials/services, DNA sample quantification, etc. [76]. The four basic steps of library preparation are as follows: DNA fragmentation, adapter sequences, size selection, and final library quantification and QC [75]. In essence, DNA library preparation entails converting the isolated sample DNA (cDNA) into a collection of DNA fragments, all of which are of similar size with known adapter sequences added to the 5′ and 3′ ends of each fragment [79,80]. These fragments can then be sequenced on next-generation sequencing (NGS) technology. Several companies have their own library preparation and sequencing kits, such as Illumina DNA prep (known previously as Nextra DNA Flex) and TruSeq DNA PCR-free, which is predominant in WGS studies, and both have been recognized as the industry standard; however, several alternatives are available such as Ion Torrent S5 along with Oxford Nanopore MiniON and Pacific Biosciences Sequel [76,81,82].

After DNA sequencing, the final step known as bioinformatic analysis occurs, which encompasses a wide range of computational processes, all of which help in reconstructing the microbial community within the given sample. Several approaches and methods are available, and choosing exactly which ones to use depends largely on the individual aims of the study [75,76]. However, this step usually entails quality control in which the raw output from NGS technologies is processed along with the removal of low-quality adapters and reads. Taxonomic analysis can then be performed by analyzing the metagenomics of the sample with already known sequences via software such as DOTUR [75]. Metagenomic assembly follows and is the reconstruction of genomes from the smaller fragments of DNA segments known as reads [83]. One popular method for this assembly is known as the de Bruijn graph approach, in which each sequencing read is broken down into overlapping subsequences of a fixed length k that defines the vertical edges of the de Bruijn graph [76]. The assembler then tries to find a path through the graph to reconstruct the genomes [84,85]. Furthermore, functional analysis can be performed in which biological functions are assigned to genetic sequences, identifying genes and enzymes of particular functions. This gives researchers a powerful tool to understand complex intricacies within the microbiome [75]. Several computational pipelines, such as Dragon Metagenomic Analysis Platform (DMAP), MOCAT2, and MetaStorm, have been developed and used for this task.

### 5.2. Advantages of WGS over 16S Sequencing

In assessing the gut microbiome, 16S RNA sequencing has been the most commonly used method for sequencing and compiling data. However, WGS sequencing is an alternative approach that has increased utility compared to the 16S RNA sequencing method. While both techniques allow for the identification and classification of bacteria in the gut microbiome, the scope of WGS sequencing expands well beyond that of 16S RNA sequencing. Due to the complexity of human gut microbiota and the ability of bacteria to employ horizontal gene transfer leading to substantial genetic diversity, direct gene identification is crucial for a deeper understanding of the human microbiome [13]. The ability of WGS sequencing to predict specific bacterial genes does just that. WGS sequencing can identify significantly more species due to its increased sensitivity. Additionally, the taxa are more accurately defined at the species level with WGS sequencing [13]. Overall, WGS sequencing gives a comprehensive, accurate picture of the human gut microbiome.

This increased understanding of the human gut microbiome and more precise detection of the various microbes using WGS sequencing gives it remarkable utility in the clinical setting. Such clinical applications include novel treatments for difficult-to-treat disorders such as FMTs and probiotics for bacterial dysbiosis, understanding associations between microbiome and various pathologies, and impacts on lifestyle modifications such as diet and exercise on the microbiome and, subsequently, human health. Many significant pathologies are associated with gut microbiome alterations including Inflammatory Bowel Disease (IBD), Chronic Idiopathic Constipation, colorectal cancer, obesity, coronary artery disease, and diabetes [8,13,86,87]. Understanding these alterations in the microbiome through methods like WGS also allows for identifying risk factors that may aid in disease prevention. For example, tetracycline antibiotics for acne treatment are associated with an increased risk of Crohn’s Disease due to microbial dysbiosis [87-89].

Another valuable aspect of using the WGS method is its ability to extend beyond sequencing bacteria. Not only can WGS sequencing assess bacterial composition, but it is also able to identify different kingdoms, including viruses, protozoa, and fungi [13]. While most of the current literature focuses on bacterial microbiome and its implications for human health, the importance of other microbes, such as viruses and fungi, is becoming increasingly apparent and is vastly overlooked. For example, FMTs include viruses (mainly bacteriophages) that may be involved in the success of this therapeutic intervention [86,90]. Further, fecal virome transplantation (FVT) has shown efficacy in alleviating bacterial dysbiosis secondary to antibiotic use [86,91]. The fungal mycobiome is also important in the pathogenesis of certain human gut disorders. More specifically, reduced fungal diversity and dysbiosis are associated with the development of IBD [86,92]. Therefore, the ability of WGS to identify species beyond bacteria allows for great potential in manipulating and analyzing microbiota to understand the human gut microbial community and its implications on human health.

### 5.3. Efficacy/Accuracy of WGS

Whole-genome sequencing provides a method for analyzing the complete genetic makeup of microorganisms yielding a more precise clarification of taxonomic identification, functional analysis, and a more accurate understanding of the interactions of the microbial community. Due to the comprehensive usage of the entire set of genetic material including genes and non-coding regions, the potency of this sequencing procedure relies on the multiple steps from DNA extraction to sequencing analysis to be carried out with advanced bioinformatics tools.

The accuracy of whole-genome sequencing is correlated with its ability to uncover a multitude of information that is not easily accessible using other sequencing methods. By presenting an extensive view of the community, this method allows researchers to analyze microbial diversity, interactions, and pathways and rebuild complete genome sequences [73]. An example of the possibilities of this sequencing approach has been demonstrated in a study performed on the Sargasso Sea community, an intricate population of almost 1800 species. Through complex amounts of sequencing (1.6 Gbp), WGS sequencing was able to ascertain over 1.2 million new genes and 148 new species [65,93]. While numerous studies on this specific community have existed, the impact of WGS sequencing lies in its ability to uncover microbial diversity that may not have been discovered previously. In studies using 16S rRNA in conjunction with WGS sequencing, the accuracy of WGS sequencing can be demonstrated through the method’s ability to make information available about the genome not accessible through other sequencing techniques. In a study carried out on genome sequences of the Lactobacillus population in gut microbiota, a multiple time-point analysis demonstrated the ability of WGS sequencing to identify populations that were not able to be retrieved by other commonly used methods, such as 16S rRNA sequencing. Due to the small number of lactobacilli present in fecal human samples, the libraries used by other sequencing methods are unable to demonstrate the presence of certain microbiota [94]. WGS sequencing, however, is able to accurately provide a taxonomic framework for microbes at species and strain levels [95]. In the Lactobacillus study, 58 different species were determined, with *L. rhamnosus*, *L. ruminis*, *L. delbrueckii*, *L. plantarum*, *L. casei,* and *L. acidophilus* being the most present in the microbial community, along with 86 Lactobacillus strains being identified. Whole-genomic shotgun sequencing’s ability to recognize new species was also demonstrated with 16 species being retrieved for the first time in human feces [95].

While multiple studies have demonstrated the ability of WGS sequencing to efficiently identify microbial species, the accuracy of WGS sequencing relies on standardized bioinformatics infrastructure and continued advancements in the field of genomics [96].

## 6. Advantages and Disadvantages of WGS vs. 16S Sequencing

There are both advantages and disadvantages of utilizing WGS sequencing in the identification of the gut microbiome when compared to other sequencing methodologies. Identifiable benefits of whole-genome shotgun sequencing include its enhanced detection of bacteria at the species level, increased detection of diversity, and ability to sequence other microbes such as viruses, protozoa, and fungi [13]. On the contrary, known disadvantages include its cost to run and more extensive database requirements with higher coverage [13,97]. Whole-genome shotgun sequencing has enhanced the detection of bacteria at the species level when compared to the other common modality of gene sequencing known as 16S RNA. A study by Ranjan et al. compared the species abundance detected with various read lengths between WGS and 16S RNA sequencing. At 32.8 million reads, 16S RNA detected only 1800 species compared to more than 3000 species detected by the WGS sequencing method. Furthermore, when looking at Proteobacteria, there were 1056 species only detected by WGS and not 16S RNA sequencing, proving its increased sensitivity to species detection [13]. Whole-genome shotgun sequencing has increased the detection of diversity when compared to the 16S RNA sequencing methodology. This is significant due to horizontal gene transfer in bacteria and its ability to generate a diverse genome. When utilizing different metrics of diversity analysis, such as the Shannon diversity, Simpson index, and evenness, all three measures of diversity were higher for the WGS than the 16S RNA sequencing [13]. The last major advantage of whole-genome shotgun sequencing is its ability to identify organisms in other kingdoms, such as protozoa, fungi, and viruses. One recent study by Su et al. utilized whole-genome sequencing for the SARS-CoV-2 virus to compare the complete genome of the virus. The WGS measured viral samples collected from Wuhan, China, and Singapore, Japan, to confirm the high similarity between viruses and determined Singapore’s virus likely originated in Wuhan [98]. Whole-genome shotgun sequencing is important for infectious disease monitoring as obtaining an accurate full-length sequence of the causal infective organism is important for phylogenetic or surveillance studies and taxonomy assignment, especially for fast-evolving RNA viruses [99].

On the contrary, one pitfall of WGS is the associated cost of the sequencing. Since the whole genome is sequenced, it must be analyzed and stored to determine the significance of each variant and referenced with those currently determined to be significant [100]. Genetic variants need to be validated with Sanger sequencing and many variants in non-coding regions may or may not be relevant. As a result, there is an increased cost compared to 16S RNA sequencing due to the processing, storage, and interpretation of the whole genome. However, increasing technological advances and newfound uses for WGS have drastically reduced costs. Furthermore, similar genome sequencing called whole-exome sequencing (WES) can be an order of magnitude less expensive than WGS to achieve an approximately equivalent breadth of coverage of protein-coding exons. These reduced costs offer the potential to greatly increase sample numbers, which is a key factor for many studies [101]. The depth of coverage required for WGS is another disadvantage when compared to 16S RNA sequencing. Coverage is the average number of times each nucleotide is sequenced given a set number of reads with a given length [101]. Although WGS was successful in identifying more species of bacteria than 16S RNA sequencing, it requires a higher coverage to identify and understand the surplus species identified [13]. While whole-genome shotgun sequencing enhances numerical and diversity detection at the species level and can sequence kingdoms other than bacteria, certain drawbacks remain in terms of the higher cost and increased coverage required when compared to 16S RNA sequencing. Nonetheless, WGS is an invaluable resource with great future potential in epidemiology and the gut microbial.

## 7. Discussion

The primary aim of this review was to explore the usage of 16S rRNA and WGS sequencing and compare the advantages and disadvantages of the two methods (Table 1). While both methodologies have their practical uses in laboratory and clinical settings, choosing between the options should come down to the specific objectives and resources available in the laboratories. 16S sequencing’s advantages over WGS sequencing mostly center around convenience and accessibility. Both sequencing methods can identify non-culturable bacterial species; however, 16S sequencing is more accessible through its low cost [59] and shorter run and identification times [36]. Laboratories with more specific research goals that need broad and detailed identification of different species should consider WGS sequencing instead. 16S sequencing has shown difficulties in delineating bacteria beyond the genus level [36,57,61]. Durazzi et al. utilized chicken guts to compare the sequencing data between 16S and WGS sequencing. They reported that 16S recovered less than 50% of the phyla that WGS sequencing identified, showing that 16S sequencing has very limited differentiating power at any level beyond phylum [102]. Other studies have reported different findings of how well 16S sequencing can differentiate organisms at the genus and species level [61-63]; however, these studies are older, and databases were more limited then. Because 16S sequencing only looks at the 16S sequence, any similarities between different organisms’ 16S nucleotide sequences limit the differentiating power of this sequencing method. Where 16S sequencing has its most practical usage is when standard phenotypic analyses, such as cultures, are not sufficient and when resources and costs are a constraint.

WGS sequencing is more difficult to use as a tool for assessing gut microbiota in settings with limited resources. It is a more costly analysis, which makes 16S sequencing more attractive in that regard [100]. However, if the resources are available to utilize WGS sequencing, it is better than 16S sequencing at unearthing the diversity of microbiota samples. Previous studies have highlighted the superior taxonomic resolution of WGS metagenomics. Laudadio et al. analyzed human stool samples with 16S and WGS sequencing and found that WGS sequencing was significantly more effective at assessing microbiota at the species level. WGS differentiated around three times more species from a given sample than 16S sequencing [103]. Furthering this point, Campanaro et al. found that 16S sequencing led to some underestimation bias of certain phyla, while it also overestimated some due to differences in hypervariable regions of the 16S rRNA sequence [104]. From this, it is reasonable to conclude that WGS sequencing is much more suitable than 16S sequencing for more comprehensive studies due to its superior taxonomic resolution of shotgun metagenomics.

While WGS and 16S sequencing were the primary focus of this review, they are not the only methodologies for assessing the gut microbiota. Some other methodologies that enable detailed analysis of gut microbiota include single-cell genomics and metabolomics. Single-cell genomics offers very high resolution of individual cell genomes, albeit at a significant cost. It is able to detect and connect specific functions to individual species [105,106]. Metabolomics provides insight into various metabolites and their link to health and disease. Combining metabolomics with 16S or WGS sequencing could create a comprehensive understanding of the gut microbiota and its link with pathology and various biological processes [107].

As the importance of understanding the human gut microbiota increases and its clinical relevance is appreciated, more studies need to be done assessing the validity and reliability of different sequencing methodologies—especially using human gut samples for comparing results between 16S and WGS sequencing. Over time, technology usually becomes more affordable, so the disadvantages of WGS sequencing, given its higher cost, might disappear. One methodology is not necessarily better than the other every time. As we have seen here, there are many advantages and disadvantages of each method. However, the choice of methodology in assessing gut microbiota should be multifactorial in each situation.

## 8. Conclusions

Unraveling the prevalent sociobiome allows us to map and normalize the expected microbiome of a population, and this can then be extrapolated to individual subjects. Any dysbiosis, when diagnosed, especially when attributable to disease, can be understood in relation to changes in bacterial richness and diversity, as explained by undertaking 16S rRNA sequencing and/or whole-genome shotgun sequencing. With initial experimentation, restoration of the microbiome can be attempted utilizing currently available or developing targeted prebiotics, probiotics, synbiotics, postbiotics, fecal microbial transplant, or drugs targeting relevant receptors, genes, enzymes, or microbes. While 16S rRNA is still utilized in specific situations in which only bacteria within a sample are of concern or when the cost is a prohibitive factor, it has largely been replaced by WGS sequencing, as previously mentioned. WGS sequencing allows clinicians and researchers to better understand the complex relationships between all microbes within a given sample and how they might be contributing to physiologic and disease pathologies or both. This sequencing technique has led to revolutionary medical insights which are being further explored.

## Figures and Tables

**Table 1.** Comparing variables and advantages/disadvantages of 16S Sequencing vs. WGS [13,97-101].

	16S Sequencing	WGS Sequencing
Goal	Targets 16S gene specific to bacteria and archaea	Sequences all genetic material in sample
Taxonomic Resolution	Identification to genus level; limited ability to distinguish species	Precise identification to species level
Scope	Bacteria only	All organisms present
Cost	Low cost	High cost
Time	Shorter run time	Longer run time due to volume of data
Bioinformatics	Relies on extensive 16S rRNA databases for bacterial identification	Relies on more extensive and sophisticated databases for comprehensive analysis
Same size requirements	Smaller sample sizes	Larger sample sizes
Sensitivity	Less sensitive for species diversity	More sensitive for species diversity
Applications	Identifying and comparing bacterial communities in microbiome samples	Detailed identification and analysis of complete microbiome samples
Advantages	Cost effective, short run time, identifies non-culturable bacteria	Comprehensive detection of microbiome diversity, high resolution, extends beyond bacterial identification
Disadvantages	Limited scope and resolution	Higher cost, longer run time, extensive resource requirements
